# Novel educational strategies to improve the telemedicine clinical skills of medical students

**DOI:** 10.1017/S1463423625000040

**Published:** 2025-02-03

**Authors:** Judith Greengold, Harisa Spahic, Janet Serwint, Sharon Dlhosh, Lili Barouch, Karina Gattamorta, Amit Pahwa, Helen Hughes

**Affiliations:** 1 Johns Hopkins University School of Medicine, Baltimore, USA; 2 University of Colorado School of Medicine, Aurora, USA; 3 University of Miami School of Nursing and Health Studies, Coral Gables, USA

**Keywords:** Coaching, extraneous, innovation, medical education, simulation, telehealth, telemedicine

## Abstract

**Aim:**

Test educational interventions to increase the quality of care in telemedicine.

**Background:**

Telemedicine (TM) has become an essential tool to practise medicine around the world. However, education to address clinical skills in TM remains an area of need globally across the health professions. We aim to evaluate the impact of a pilot online learning platform (OLP) and standardized coaching programme on the quality of medical student TM clinical skills.

**Methods:**

A randomized pilot study was conducted with fourth-year medical students (n = 12). All participants engaged in video-recorded standardized patient (SP) simulated encounters to assess TM clinical skills before and after the intervention. Participants were randomized to either the OLP or OLP + Virtual Coaching Institute (VCI) intervention cohort. Quantitative and qualitative data were collected to address self-reported skills, attitudes, and self-efficacy before the 1st SP encounter and after the 2nd SP encounter. SP encounter recordings were scored by two blinded non-investigator raters based on a standardized rubric to measure the change in TM care delivered pre- and post-intervention. Statistical analysis of quantitative data included descriptive statistics and mixed effects ANOVA.

**Findings:**

Recruitment and retention of participants exceeded expectations, pointing to significant enthusiasm for this educational opportunity. Self-reported skills and scored simulation skills demonstrated significant improvements for all participants receiving the interventions. Both OLP and VCI interventions were well received, feasible, and demonstrated statistically significant efficacy in improving TM clinical skills. Participants who received coaching described more improvements in self-efficacy, confidence, and overall virtual clinical skills. This study provides evidence that virtualized clinical learning environments can positively impact the development of TM clinical skills among medical students. As TM continues to evolve, the implementation of innovative training approaches will be crucial in preparing the next generation of healthcare professionals for the demands of modern healthcare delivery.

## Introduction

The landscape of telemedicine (TM) clinical skills education remains an evolving frontier in the wake of the transformative impact of the COVID-19 pandemic on the accessibility and scope of virtual care (Hayes-Roth *et al*., 2009; Szyld *et al*., 2017; Sasnal *et al*., 2021; Villegas *et al*., 2021). As the global TM scope has grown, so too have the recommended competencies for health professions students to meet the needs of this emerging medium of clinical diagnostic reasoning (AAMS, 2020). Recognizing the pressing need to clarify the strengths and limitations of virtual clinical examination settings will help guide future educational efforts and define clinical best practices as TM deployment continues to evolve.

Research has highlighted significant gaps in clinician knowledge of virtual clinical examination strategies. Recent surveys of primary care providers found that fewer than 40% of providers felt confident in their ability to conduct complete TM physical exams while more than 40% of providers reported a strong need for education on TM clinical skills (Greengold *et al*., 2021); this gap has notably persisted even among TM superusers as of 2022 (Greengold and McGuire, 2025). As health professions educators around the world worked rapidly to address the challenge of providing high-quality educational experiences for pre-professional learners in virtualized learning environments during the COVID-19 pandemic, much remains unknown about unique strengths and challenges of maintaining quality in virtual clinical learning environments and best practices in training the future workforce around the world (Ghaddaripouri *et al*., 2023). International studies have found general acceptance of TM as a valuable tool among learners (Ghaddaripouri *et al*., 2023), applying global standards in medical education remains a significant gap to address this clinical skillset (Bajra *et al*., 2023).

This pilot study aimed to identify clear strategies for TM clinical skills education with the goal of improving the quality of virtual care for patients and enhancing learning opportunities for health professions students in virtualized clinical learning environments. Both coaching and simulation have successfully improved learner confidence and assessed clinical skills when done in person for traditional face-to-face patient interactions (Ravitz *et al*., 2013; Moazed *et al*., 2013; Soucisse *et al*., 2017; Lovell, 2018). Given the success of these interventions in the traditional format, we hypothesized that these interventions, especially in combination, will be successful in virtual learning environments.

This innovative study will be the first to our knowledge to examine how TM clinical skills training can improve the quality of virtual care by evaluating the impact of virtually delivered one-on-one coaching and traditional online learning methods. We hypothesize that targeted educational offerings (online learning platform (OLP) ± a virtual coaching session (VCI)) will enhance the quality of TM clinical skills observed in standardized patient (SP) encounters. We further hypothesized that participants receiving the VCI intervention would have greater increases in their quality of the TM clinical skills observed in SP encounters as compared to participants who only received the OLP. This pilot study sought to understand the student learner perspectives of these educational experiences and explore the feasibility of larger-scale implementation of these interventions.

## Methods

### Participants

**Figure 1.**
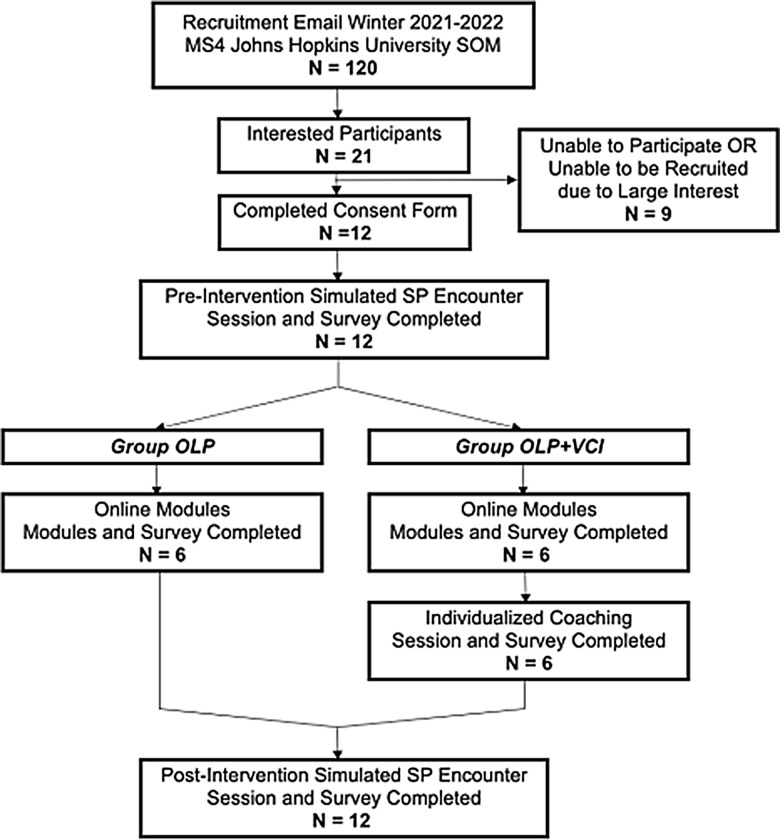
PRISMA diagram of study design and methods.

The study enrolled fourth-year medical students from Johns Hopkins University School of Medicine who had successfully completed all core clinical rotations (Figure 1). Recruitment took place in the winter of 2021 through email invitations. The sole exclusion criterion was the prospective participant’s inability to fulfil all aspects of the study. All participants provided informed consent and received remuneration for their time in the amount of $25 electronic gift card per completed phase of the project for a total of $75 per participant. This study was reviewed and approved by the Johns Hopkins University School of Medicine Institutional Review Board.

### Study design

**Figure 2.**
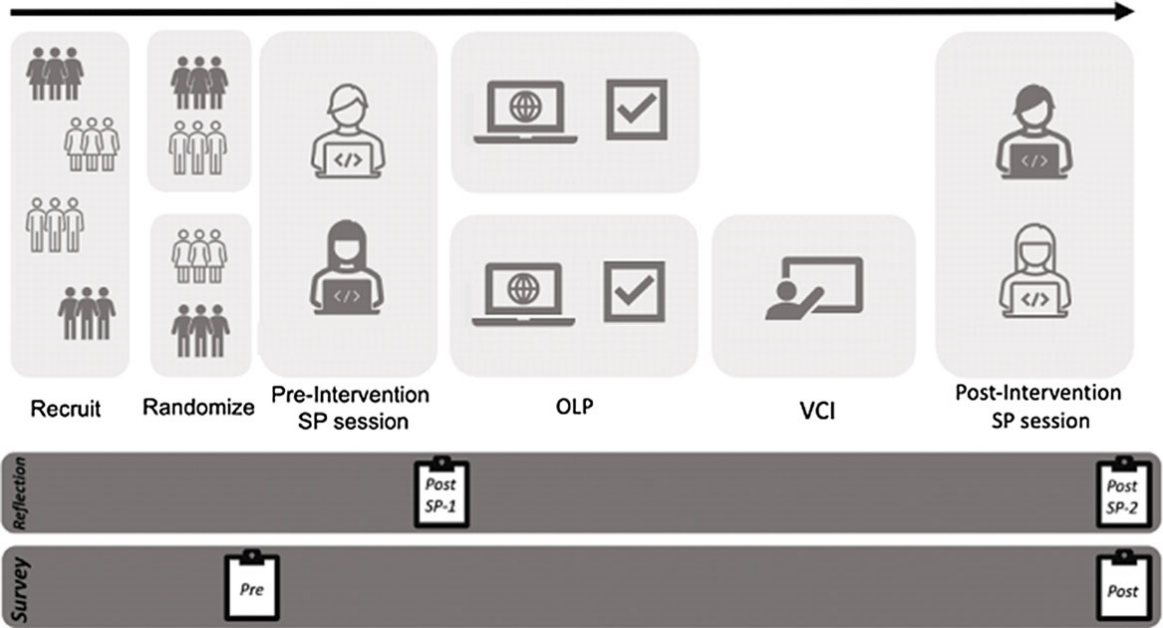
Study design.

Chronologically, this study timeline consisted of a Pre-Study Survey, 1st SP encounter, Post-SP Participant Self-Reflection, Intervention, 2nd SP encounter, Post-SP Participant Self-Reflection, and Post-Study Survey (Figure 2). By using SP encounters with only one SP, we aimed to control relevant medical information to assess these outcomes without risking harm to patients and reducing variability between learners. Two different cases utilizing abdominal pain as the chief complaint were used during the SP encounters. Participants were independently randomized to case order, survey question set order, and intervention.

### SP encounters

The SP encounters were 15 minutes long and recorded. The SP was a retired physician with five years of experience as an SP at the Johns Hopkins University School of Medicine Simulation Center. Students received a brief past medical history approximately four hours in advance and vitals sheets around five minutes before the scheduled session. Acting as the primary care physician, students were tasked with conducting a comprehensive encounter including history, a virtual focused physical exam, and sharing their assessment and plan with the SP.

During the videoconferencing session, technical challenges were deliberately introduced to assess students’ ability to troubleshoot and resume the TM visit with full audio and video connection. The encounters were timed and after 15 minutes, the research assistant concluded the session. Following each encounter, students were prompted to immediately complete a Post-SP Self-Reflection. In this reflection, participants reported their confidence in the encounter, shared insights into successful elements and challenging moments, and provided additional comments about their overall experience. This structured approach aimed to comprehensively assess students’ TM clinical skills and their capacity to navigate technical challenges in a simulated clinical setting.

### Intervention

All participants completed the online learning platform (OLP). Half of the participants went on to complete the Virtual Coaching Institute (VCI) intervention. Consequentially, the study had two cohorts: ‘OLP’ and ‘OLP+VCI’.

#### Online learning platform (OLP)

**Figure 3.**
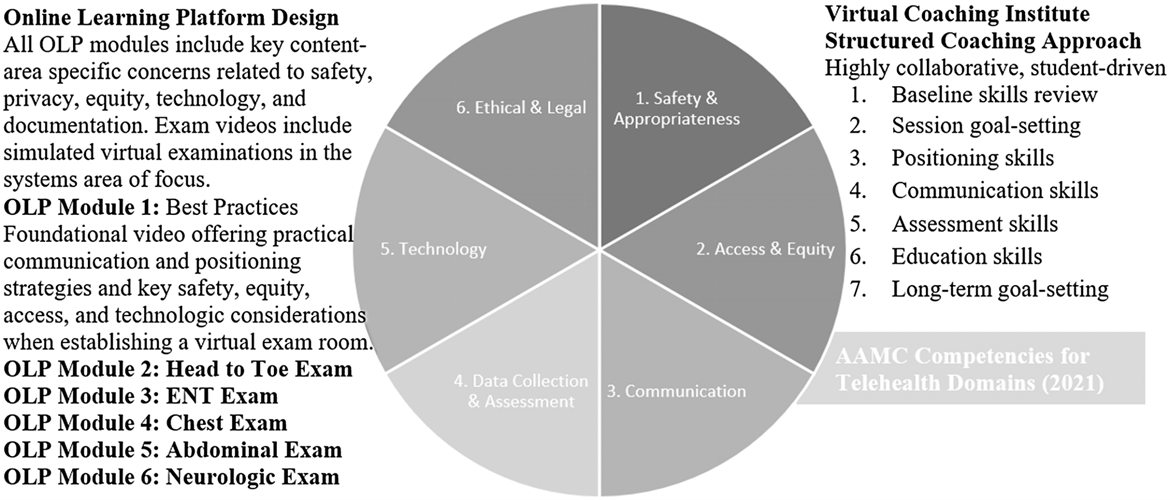
Intervention content in alignment with AAMC competencies for telehealth domains (2021).

The Johns Hopkins Telemedicine Education Consortium Clinical Skills OLP contains 6 videos of clinical skills demonstrations. Content drew on AAMC Competencies for Telehealth (2021) and reflected key objectives concerning safety, privacy, access, equity, and technology usage (Figure 3). The total time to watch all videos was approximately 75 minutes. Participants received an email with links to all six videos from the research assistant once they completed the 1st SP session and Post-SP Reflection. Once students stated they had completed the OLP, they were directed to complete the next steps in their cohort. OLP participants proceeded to their 2nd SP encounter, while participants in the OLP+VCI cohort proceeded to their coaching session (VCI) and then went on to complete the 2nd SP encounter. After the 2nd SP encounter, participants were directed to complete post-intervention and post-SP encounter surveys. All surveys were completed among all participants.

#### Virtual Coaching Institute (VCI)

The VCI consisted of a video-recorded 30-minute individualized coaching session with an expert primary care physician and faculty member from the Johns Hopkins Telemedicine Education Consortium, experienced in developing the curriculum for the Clinical Skills Curriculum OLP. The coach received a copy of the student’s Pre-Study Survey and Post-SP Self-Reflection but not the recording of the SP simulated encounter. Students were also sent a copy of their Post-SP Self-Reflection prior to the coaching session and were asked to identify areas of TM skills they would most like to focus on during the session. The coach followed a structured session guide which included pre-assessment, goal setting, a skills exercise, and feedback (Figure 3). Given the coach’s expertise in primary care and TM clinical skills, the coach was given the option to extend the coaching session beyond 30 minutes if needed.

### Surveys

The Pre- and Post-Study Surveys were developed using the Kirkpatrick Model methodology to reflect questions assessing Levels 1 through 4 of Learning Evaluation. Likert scales were employed to assess participants’ self-reported skills, attitudes, and self-efficacy. The Pre-Study Survey additionally collected demographic data about participants and previous experiences within TM. The Post-Study Survey further assessed students’ ratings of the OLP and VCI. All surveys were collected through the secured Qualtrics platform.

### SP encounter scoring

A detailed rubric was developed to evaluate clinical skills and communication domains in consultation with instructional designers from the Johns Hopkins School of Medicine Office of Assessment and Evaluation. Each SP interaction video was scored by two independent non-PI reviewers utilizing a standardized rubric assessing key components of the interaction (e.g. communication, key portions of the history, physical exam manoeuvres, rapport, and TM particularities). Reviewers were blinded to the pre- and post-intervention status of the video-recorded simulation. If a difference in scoring arose between reviewers, the difference was rectified by review and agreement from a third scorer.

### Data analysis

Evaluations from pre- and post-SP encounters were numerical scores assessed as changes in scores pre- and post-intervention. Survey data utilized Likert scales. Subdomains were totalled and averaged, approximating continuous variables. SP Encounter scoring data was further analyzed through descriptive statistics and logistic regression. Complex mixed ANOVA assessing changes in domain, time, and group found no significant differences per group so two-way repeated measures ANOVA was run with domain and time as the two within-subjects variables. Further analysis was run using SPSS Statistical Software version 28. Analytic methods were reviewed in consultation with a data analysis librarian and biostatistician.

## Results

Participant characteristics of this pilot study are found in Table 1. Notably, most participants were female and represented a wide variety of residency specialties to which they matched. Over half of students (N = 7) reported no or low TM experience and few described interest in primary care-oriented specialties.

**Table 1.** Participant demographics

Cohort	OLP (n = 6)	OLP + VCI (n = 6)
Sex (F, %)	3 (25%)	6 (50%)
**Intended residency**		
Ophthalmology	0 (0%)	2 (17%)
Dermatology	2 (17%)	0 (0%)
Neurology	1 (8%)	0 (0%)
Pathology	0 (0%)	1 (8%)
Emergency medicine	1 (8%)	0 (0%)
Radiation oncology	1 (8%)	0 (0%)
Medicine-Paediatrics	0 (0%)	1 (8%)
Paediatrics	0 (0%)	1 (8%)
N/A	1 (8%)	1 (8%)
**Interest in primary care**		
**Low**	**6 (50%)**	**2 (17%)**
Medium	0 (0%)	2 (17%)
High	0 (0%)	2 (17%)
**Previous telemedicine experience**		
None	0 (0%)	1 (8%)
Low (<5 visits)	4 (33%)	2 (17%)
Moderate (6–10 visits)	2 (17%)	2 (17%)
**High (>10 visits)**	**0 (0%)**	**1 (8%)**

### Self-reported confidence levels

There was an overall statistically significant increase in all participants’ self-reported confidence across nearly all domains after the intervention (Table 2, Figure 3). Sphericity was not violated, so repeated measures of ANOVA analysis were conducted. All participants improved in Overall Confidence (*p* < .001), Setting up the Encounter (*p < .*001), Clinical Examination (*p* = .081), and Communication (*p* = .008). Participants who received coaching in addition to the online videos (OLP+VCI) had on average greater confidence in more domains compared to students in the OLP-only group and were more likely to describe marked improvements in confidence. Marked improvement was described in the domains of Setting up the Encounter and Clinical Examination in the OLP+VCI. All domains demonstrated statistically significant improvement with the exception of history-taking, which was notably high for both groups at baseline and subsequently demonstrated only mild improvement across both groups.

**Table 2.**
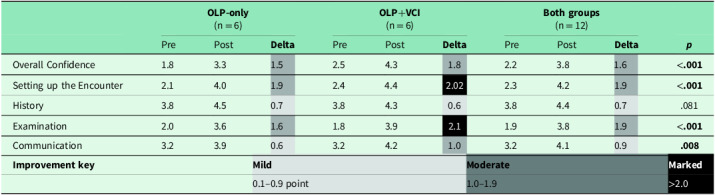
Changes in self-reported confidence from pre- to post-interventions: online learning platform (OLP) and Virtual Coaching Institute (VCI)

### Student’s rating of intervention

Participants had overall positive reviews of both the OLP and VCI (Table 3). While students in the OLP+VCI had overall more highly positive reviews compared to the OLP-only group, all students rated the interventions as helpful and appropriate in length and described a high likelihood to recommend the interventions to friends. Interestingly, students in the OLP+VCI group indicated having both OLP and VCI improved their TM skills (2.5 on a 5-point Likert scale) compared to OLP alone (2.0 on a 5-point Likert scale) or VCI alone (2.3 on a 5-point Likert scale).

**Table 3.** Intervention self-reported attitudes online learning platform (OLP) (n = 12) and Virtual Coaching Institute (VCI) (n = 6)

	Agree/strongly agree	Disagree/strongly disagree
Overall Helpfulness of OLP: Helpful	100%	
Likelihood to Recommend OLP to Friend: High	100%	
Length of OLP: Appropriate	100%	
OLP Level Relative to Training: Appropriate	100%	
Impact of OLP Alone on Clinical Skills: Improve	100%	
Overall Helpfulness of VCI: Helpful	100%	
Likelihood to Recommend VCI to Friend: High	83%	1 (17%)
Length of VCI: Appropriate	83%	1 (17%)
VCI Level Relative to Training: Appropriate	100%	
Impact of VCI Alone on Clinical Skills: Improve	100%	
Impact of VCI+OLP on Clinical Skills: Improve	100%	

Qualitative responses allowed students to comment directly and convey feedback in their own words. Themes about reactions to the OLP included ‘*the length and content were really good – they were efficient and got right to the point of the important components of a telemedicine exam*’ and appreciation for ‘*example demonstrations with a simulated patient*’ while providing recommendations for improvements such as considering ‘*a written transcript*’ and ‘*one concise video*’. One participant in the OLP-only group elaborated that ‘*actual practice with a telemedicine encounter would help further cement the telemedicine skills into my mind*’. Participants in the OLP+VCI cohort described the coaching sessions as including ‘*personalized feedback*’, ‘*specific to my goals*’, and valued the opportunity to have ‘*feedback in real time*’.

### Review of participant telemedicine skills

Analysis of the SP Simulated Encounter illuminated challenges in utilizing a large format assessment tool completed by multiple scorers. Although all scorers received the same recorded training and standardized form vetted extensively by educational design experts, response discrepancies were noted between reviewers. The areas of strongest concordance were in the areas that participants failed to examine; the HEENT exam, Chest Exam, and Failure to verify backup methods for maintaining the visit were the three subdomains without discrepancies. The initial scoring scale of 1 to 4 was simplified to a pass/fail score represented by a 0 or 1 to reflect scores selected below 3 (failed skill) or above 2 (passed skill). The interrater reliability of the dichotomized scale was 88%. We then analyzed the two primary domains of Communication and Examination. No significant differences were noted between the groups (*F*(1, 10) = .05), *p* = .822). While no domain-by-time interaction effect was found (*p* = 0.18), the main effect of domain and time was significant (*F*(1, 10) = 5.002, *p* =.049), confirming the significant improvement in Communication and Examination skills after the intervention. Despite the small sample size for the pilot study, these findings highlight a positive and significant improvement in virtual clinical skills with the virtual clinical skills educational interventions.

## Discussion

This pilot study aimed to deploy and evaluate high-quality educational experiences to improve the diagnostic abilities of medical students caring for patients via TM. Providing formalized TM clinical education was well accepted, feasible, and effective at improving the skills, knowledge, attitudes, and self-efficacy of medical students. Participants were eager to engage with a TM clinical skills intervention regardless of specialty and represented a diverse range of clinical interests and backgrounds. No participants left the study or were unable to complete the various items. Feedback from students echoed a sense of appreciation for the materials presented and offered suggestions for future iterations of the intervention. Overall, students described a perception of skill improvement, and this was demonstrated in the analysis of clinical skills domains from the SP encounters.

### Impact of coaching

Although the study results may not show a significant benefit/value in the addition of personalized coaching for TM skills improvement, several students commented on the benefit of receiving tailored feedback on their video presence and virtual examination and communication skills. One participant described feeling especially ill-prepared for and less confident in anticipated TM interactions with actual patients; she exclaimed that the coaching she received in the intervention would go a long way to boost her confidence and give her a grounded presence and voice of authority as she prepares to communicate important pathological findings with her patients during residency. Listening to and amplifying the voices of students has helped guide and shape this research and future directions for tailored educational interventions.

### Impact of COVID-19 pandemic

The COVID-19 pandemic likely played a unique role in the delivery of the study; students were especially interested in TM utilization given the urgency of access to TM in a resource-limited setting. Moreover, clinical experiences for students during this time period were significantly impacted by clinic site restrictions and higher dependence on simulated encounters. This study also represented a unique opportunity for students to engage in medical research from a remote setting, enabling them to access research from the comfort of their homes and without the traditional challenges of transportation, parking, and time management with a busy student schedule. COVID-19 pandemic-related challenges in healthcare settings significantly restricted faculty and resource availability in the analytic phase of the study, delaying scoring and data analysis.

### Limitations

There are a number of limitations that should be considered when interpreting these results. The small sample size limited generalizability, but given that statistical significance was noted in multiple domains, it offers an important starting point for future interventions. While we did not find that the coaching intervention would yield stronger results than the OLP in isolation, this may reflect the small sample size and the challenges in scoring. The scoring tools were developed *de novo*, and their usability could be improved in future iterations. The diversity of professional interests among participants provided both a neutralizing factor and perhaps an important confounder to consider in future studies; limiting participant recruitment to those with the strongest interest in proceeding to a career that includes a high likelihood of TM integration may help identify unique strengths and limitations of this content when deployed among people most likely to utilize the lessons in clinical practice.

### Future directions and practical implications

This pilot study helps shine a light on current gaps and opportunities in traditional medical education as well as important areas for refinement in methodology and content for future iterations of the Johns Hopkins Telemedicine Education Consortium clinical education offerings. Importantly, a large discrepancy between a student’s self-reported skills and observed skills in SP encounters was noted; adding direct observation prior to the coaching intervention may enhance the quality of the coaching intervention and avoid biasing the goal-setting period of the VCI. Though it is clear that TM clinical educational tools are desired, appreciated, and effective, further work is needed to clarify the role of coaching in developing health professions students’ clinical competencies. Considering implementing a pre-coaching review of the simulated encounter may arguably change the coach’s approach to the session flow. Lessons learned can enhance future studies and educational interventions.

Importantly, this research highlights both the need and the feasibility of augmenting learner and clinician education by utilizing technology to enhance the care environment. The study design can be implemented in academic as well as professional settings to support learners and clinicians who may be interested in improving this important skill set. Moreover, the research underlines a need to clarify the relationship between the virtual physical examination and diagnostic accuracy; utilizing a range of cases and presentations and increasing the number of participants may yield a clearer picture of the impact of education on diagnostic accuracy in the virtual setting.

## Conclusion

The pilot study identified gaps and improvement opportunities in the traditional medical education curriculum for teaching TM skills and the Johns Hopkins Telemedicine Education Consortium educational materials. The experiences underscore the importance of providing coaching opportunities to improve and enhance the efforts and skillsets of those learners who may fall short of the desired competencies. Virtually led recruitment and intervention deployment were demonstrated to be logistically feasible and effective for launching a complex and dynamic clinical skills education opportunity for medical students. Lessons learned underscore important educational approaches to improving healthcare quality and safety. More research is needed to determine the role of coaching in developing clinical competencies as well as the impact of TM education on diagnostic accuracy in virtual settings.
